# Lifestyle Trajectories Are Associated with Incidence of Cardiovascular Disease: Highlights from the ATTICA Epidemiological Cohort Study (2002–2022)

**DOI:** 10.3390/life13051142

**Published:** 2023-05-08

**Authors:** Evangelia Damigou, Matina Kouvari, Christina Chrysohoou, Fotios Barkas, Evrydiki Kravvariti, Christos Pitsavos, John Skoumas, Evangelinos Michelis, Evangelos Liberopoulos, Costas Tsioufis, Petros P. Sfikakis, Demosthenes B. Panagiotakos

**Affiliations:** 1 Department of Nutrition and Dietetics, School of Health Sciences and Education, Harokopio University, 17676 Athens, Greece; 2 First Cardiology Clinic, Medical School, National and Kapodistrian University of Athens, Hippokration Hospital, 15772 Athens, Greece; 3 First Department of Propaedeutic Internal Medicine, Medical School, Laiko General Hospital, National and Kapodistrian University of Athens, 15772 Athens, Greece

**Keywords:** lifestyle trajectories, cardiovascular disease risk, cardiovascular disease incidence, epidemiology, prevention

## Abstract

The study aimed to assess the trajectories of lifestyle characteristics and their association with 20-year cardiovascular disease (CVD) incidence. In 2002, 3042 Greek adults (aged: 45 (12) years) free of CVD were enrolled. In 2022, the 20-year follow-up was performed on 2169 participants; of those, 1988 had complete data for CVD. The 20-year CVD incidence was 3600 cases/10,000 individuals; the man-to-woman ratio was 1.25, with the peak difference in the 35–45 age group (i.e., 2.1); however, a reversal of the trend was observed in the age-groups 55–65 and 65–75, with a resumption of an almost equal incidence in those >75 years. In multi-adjusted analysis, age, sex, abnormal waist circumference, hypercholesterolemia, hypertension, and diabetes were positively associated with 20-year CVD risk, explaining 56% of the excess CVD risk, whereas an additional 30% was attributed to lifestyle trajectories; being physically active throughout life-course and being close to the Mediterranean diet were protective, while continuous smoking was detrimental against CVD risk. Mediterranean diet adherence protected against CVD development even if not sustained, while quitting smoking or engaging in physical activities during the 20-year observation did not offer any significant protection. A life-course personalized approach that is cost-effective and long-term sustained is needed to prevent CVD burden.

## 1. Introduction

In many European countries, age-standardised cardiovascular disease (CVD) mortality rates tend to decline, yet CVD morbidity remains high. Based on the European Society of Cardiology (ESC) Atlas of Cardiology, in 2019, 113 million people were living with CVD and 12.7 million new cases across the ESC member countries were observed [1]. Time-series data show a slow decline in the median age-standardized incidence of CVD, between 1990–2019, around 17% [1]. The disease burden is higher in middle-income countries. However, according to the Global Burden of Disease study, even in high-income countries, a rise in the previously declining age-standardized rate of CVD has been observed [2].

The increasing burden of CVD seems to be attributed to the increasing prevalence of modifiable risk factors [2,3]. Among them, lifestyle factors have a significant potential for reducing risk. Moreover, lifestyle behaviors present the highest variability affected by different conditions such as income, family status, clinical condition, and psychological health. Even since 2000, the Nurses’ Health Study revealed an impressive number of 80% preventable coronary events in the context of lifestyle choices [4]. A few years later, according to the INTERHEART study, modifiable risk factors, including obesity, smoking, physical inactivity, and unhealthy dietary patterns, accounted for the vast majority of the population-attributable risk for myocardial infarction [5]. There is a longstanding recognition that diet plays a significant role in the aetiology of many chronic diseases. It is estimated that among all behaviors, nutrition contributes the most to CVD morbidity across Europe [3]. Similar beneficial outcomes stand for a physically active lifestyle with no smoking and alcohol intake, resulting in the retention of healthy weight status and achieving better cardiometabolic health, followed by increased life expectance by around 12 years [6]. Prevention inevitably has to start as early in life as possible. Nonetheless, the question is whether the health benefits remain when introducing a healthy lifestyle even at later stages. Examination of the long-term incidence of CVD in parallel with lifestyle trends over a long period is urgently needed to understand better these factors’ role in the burden of CVD [7].

Thus, the purpose of the present study was to evaluate the association between the three major lifestyle trajectories, i.e., smoking habits, Mediterranean diet adherence, physical activity level, and 20-year CVD incidence. To achieve this, we used data from the population of Greece, a country previously considered a low CVD risk region [7] yet lately assigned to European countries with moderate CVD risk [8]. Our research hypothesis was that lifestyle modification towards cardiac-friendly choices even later in life would be beneficial, while adhering to healthy behaviors early in life might still have some benefits, even if the person did not sustain this healthy behavior throughout the lifespan.

## 2. Materials and Methods

### 2.1. Study Design

The ATTICA study is a prospective cohort study in a sample of Greek adult men and women with multiple follow-up examinations (i.e., 2001/2: baseline, 2006: 5-year, 2012: 10-year, 2022: 20-year follow-up). The main goals of the study were to record and monitor the distribution of several sociodemographic, lifestyle, clinical, biochemical, and psychological CVD risk factors at various time points, to explore the associations between the aforementioned factors and long-term CVD risk, and to evaluate the trajectories of these factors, regarding their predictive significance on CVD.

### 2.2. Setting and Participants

Participants were from Attica, Greece (78% urban municipalities, including Athens’s capital city). The initial sample included *n* = 3042 free-of-CVD individuals who agreed to participate out of *n* = 4056 invited between 2001–2002. Sampling was random and stratified by sex, age group and region, according to the 2001 census. Detailed information about the study aims, design, sampling procedure and methodology can be found in previously published papers [9,10].

### 2.3. Endpoint and Follow-Up Examination

**Figure 1:**
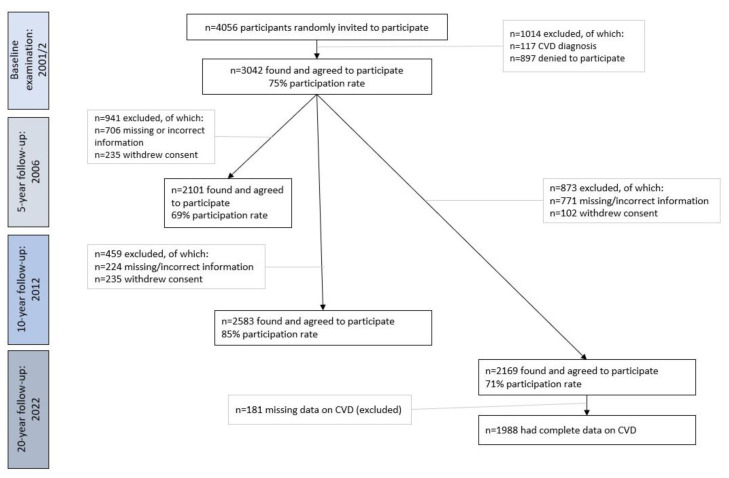
Flow diagram of the ATTICA study participants (2002–2022) included in this analysis (*n* = 1988). Abbreviations: CVD: Cardiovascular disease.

In 2022, out of the *n* = 3042 original participants, *n* = 2169 were found and participated in the follow-up, providing written consent (71% participation rate). Of those lost to follow-up (*n* = 873), *n* = 771 were lost due to changed, missing or incorrect addresses or telephone numbers and *n* = 102 refused to be examined again (Figure 1).

The combined primary endpoint studied in this work was the development of a fatal or non-fatal CVD event, defined according to the World Health Organization (WHO)–International Coding Diseases (ICD)-10 criteria [9]. For deceased participants, information was retrieved from relatives and death certificates.

For the scope of the present work, *n* = 1988 participants with complete CVD evaluation in all follow-up assessments were used. No differences were observed in the age-sex distribution of this working sample and the baseline (*p*-values > 0.80).

### 2.4. Bioethics

The ATTICA study was carried out in accordance with the Declaration of Helsinki (1989) of the World Medical Association and was approved by the Institutional Ethics Committee of Athens Medical School (#017/1.5.2001). All participants were informed about the aims and procedures and agreed to participate, providing written consent.

### 2.5. Baseline Assessment

During baseline assessment, all participants were physically examined by a trained physician. A detailed medical history containing information about cardiovascular risk factors, the use of relevant medications and a family history of cardiometabolic diseases was obtained. Baseline assessment included sociodemographic, anthropometric, lifestyle, clinical and biochemical measurements [9].

#### 2.5.1. Socio-Demographic Characteristics

The sociodemographic characteristics that were evaluated in this paper were age and sex.

#### 2.5.2. Anthropometric Measurements

Body weight, height and waist were measured following standard procedures, and body mass index (BMI) was calculated as weight/height^2^. Increased waist circumference (WC) was defined as ≥102 cm for men and ≥88 cm for women [11]. Participants were classified as overweight (25 ≤ BMI < 30 kg/m^2^) or obese (BMI ≥ 30 kg/m^2^) [12].

#### 2.5.3. Biochemical Measurements and Clinical Characteristics

Information about biochemical measurements can be found elsewhere [9]. Regarding clinical characteristics, hypercholesterolemia was defined as total cholesterol > 200 mg/dL (or reception of medication), hypertension as systolic blood pressure (SBP) ≥ 140 mmHg or diastolic blood pressure (DBP) ≥ 90 mmHg (or medication), type 2 diabetes mellitus (hereinafter reported as diabetes): fasting blood glucose ≥ 126 mg/dL (or use of insulin and/or oral hypoglycemics).

#### 2.5.4. Lifestyle Characteristics

Dietary assessment was based on a validated semi-quantitative food frequency questionnaire [13]. Overall dietary quality was defined according to the level of adherence to a Mediterranean-type diet via MedDietScore (which ranges from 0 to 55) [14]. Tertiles of MedDietScore were calculated, and comparisons were made between those in the low adherence group and those in the merged group of medium/high adherence. To assess participants’ physical activity level, the short-form International Physical Activity Questionnaire (IPAQ), which is validated for the Greek population, was used [15]; comparisons were made between those who were inactive versus those who were active (i.e., minimally/highly active, according to MET-minutes/week) [16]. Current smokers were defined as participants who smoked (≥1 cigarette/day), former smokers: who had ceased smoking (≥1 year prior), and never-smokers: with no history of smoking. Pack-years of cigarette smoking were calculated for each participant by multiplying smoking duration (in years) with the number of packs/day (assuming 20 cigarettes in a pack).

### 2.6. Trajectories of Lifestyle Parameters

Lifestyle parameters (i.e., diet quality, physical activity level and smoking status) were repeatedly assessed in 5-, 10- and 20-year follow-up periods. From baseline examination in 2002 to the latest follow-up in 2022, lifestyle trajectories were defined as follows: dietary habits, as assessed through adherence to the Mediterranean diet, were defined by four trajectories: always away versus away to close versus close to away versus always close to the Mediterranean dietary pattern; four trajectories of physical activity were also defined: always inactive versus became inactive versus became active versus always active; and, trajectories of smoking habits were described as always smoking versus started smoking versus quitted smoking versus never smoking.

### 2.7. Follow-Up Assessment

Detailed clinical information was retrieved through face-to-face interviews with trained health professionals and medical records. All participants were appointed through telephone calls. They were evaluated and invited to participate in the follow-up. If participants had died during the follow-up period, information was obtained from their relatives and certified vital records. Anthropometric, lifestyle and behavioral characteristics were assessed similarly to baseline examination. Apart from participants’ vital status (death, cause of death, non-fatal CVD event), the development of conventional CVD risk factors, including hypercholesterolemia, hypertension, and diabetes, was also recorded.

### 2.8. Statistical Analysis

Crude incidence rates of combined CVD (i.e., non-fatal and fatal CHD or stroke) were calculated as the ratio of new cases to the number of participants in each follow-up. Categorical variables are presented as relative frequencies, and associations between these variables were tested using the chi-square test. Continuous variables are presented as mean values (standard deviation-SD) and were tested for normality through P-P plots. After controlling for equality of variances using Levene’s test, comparisons of mean values of normally distributed variables (between participants who developed an event versus the rest) were performed using Student’s *t*-test. For continuous variables not normally distributed (i.e., MedDietScore, pack-years of smoking), the Mann-Whitney non-parametric test was applied, and these variables are presented as median (interquartile range-IQR). The hazard ratios (HR) of developing a CVD event during the 20 years and their 95% Confidence Intervals (CI) were estimated using Cox proportional hazards analysis. The time to CVD event was recorded on an annual basis. To evaluate differences between groups of participants regarding CVD incidence, the log-rank test was also applied. Interactions between age and sex were tested and remained in the model when significant. Each of the three main exposure factors of the present analysis (i.e., trajectories of smoking, Mediterranean diet adherence, and physical activity) were studied through nested models after adjusting for known confounding factors, i.e., age, sex, as well as history of hypercholesterolemia, hypertension, diabetes, and abnormal waist circumference. We used this analysis approach to better reflect the potential additional effect of the risk of CVD (i.e., attributable risk) of the tested lifestyle trajectories. The order of the three lifestyle trajectories entered in the models was decided according to the effect size of each trajectory (from the highest to the lowest). The *Efron* approximation was used for handling ties of person-years in the final model because the time-to-event data were skewed. To evaluate the impact of risk factors on CVD incidence, the attributable fraction (i.e., the proportion of CVD cases that is attributed to a certain risk factor) was calculated using the formula [p × (HR − 1)/(1 + p × (HR − 1)]; p is the prevalence of the factor. This sample was adequate for statistical power to assess greater than 10% differences in relative risk, at a statistical significance level of less than 0.05 (*p*-value) and with statistical power > 80%, for two-way controls. All reported *p*-values were based on two-tailed hypotheses and compared to a significance level of 5%. STATA version 17 (STATA Corp, College Station, TX, USA) was used for the statistical analyses.

## 3. Results

### 3.1. CVD Incidence and Mortality at 20-Year Follow-Up

Over the 20-year follow-up period, 36% (*n* = 718) of the participants (40% males, 32% females, p for gender difference < 0.001) had experienced a fatal or non-fatal CVD event (CHD: 71.7%, stroke: 4.3%, other: 24%). Because the number of defined stroke cases was relatively small, CHD and stroke were not analyzed separately. Of the *n* = 718 CVD events, 96 were fatal (man-to-woman (CVD fatality rate) ratio = 4:1). The overall 20-year CVD mortality rate was 4.8% (7.3% for men, 1.8% for women).

As presented in Table 1, the overall man-to-woman CVD incidence ratio was 1.25, suggesting higher incidence in men compared to women (p log-rank < 0.001), with the peak difference observed in the 35–45 age group (i.e., 2.1); however, reversal of the trend was observed in the age groups 55–65 and 65–75, with a resumption of an almost equal incidence in the >75 years group.

A total of *n* = 206 participants died (20-year all-cause mortality rate: 10.4%), of which *n* = 143 (13.3%) were men and *n* = 63 (5.7%) women. The causes of death were: 46.6% CVD (of which 83% were heart attacks, 9.6% stroke and 7.4% other CVD), 18% cancer, 7% infections (of which 14% due to COVID-19), 4% accidents, 1.5% neurological diseases, 1% chronic obstructive pulmonary disease and the rest (21.9%) due to unknown reasons. No sex differences were observed regarding the pattern of mortality (*p* = 0.223).

### 3.2. Temporal Trends of Lifestyle and Clinical Features over the Past 20 Years (2002–2022)

Participants’ lifestyle and clinical trajectories within the 20-year follow-up period are depicted in Table 2. Most of the participants were overweight or obese. The prevalence of hypercholesterolemia, hypertension, and diabetes, significantly increased, irrespective of the aging of the sample. After considering the age of the participants, the prevalence of hypertension was 1.6 times, hypercholesterolemia almost two times, and diabetes more than four times higher (all *p*-values < 0.01). Concerning lifestyle habits, adherence to the Mediterranean diet remained constant during the follow-up examinations; the prevalence of smoking habits remained high (i.e., almost 4 out of 10 were smokers) but showed a significant decline only at the latest follow-up in 2022 as compared to the previous; and prevalence of low (inadequate) physical activity was high, and similar in all examinations.

### 3.3. Determinants of 20-Year CVD Incidence

Participants who developed CVD within the 20-year follow-up consisted mainly of older men, who had abnormal waist circumference, increased pack years of cigarette smoking, ΒΜΙ, as well as lower adherence to a Mediterranean-type diet (all *p*-values < 0.05) (Table 3).

### 3.4. Multi-Adjusted Analysis of 20-Year CVD Incidence and Attributable Risk

As the aforementioned findings were prone to residual confounding, a multi-adjusted analysis was performed; beyond age and sex, which are well-established confounders in CVD epidemiology, medical history of hypertension, hypercholesterolemia, and diabetes, as well as waist circumference (which outperformed body mass classes in our models) were included in the core models (see model 1 & 2, Table 4). Increased age and male sex had a consistent and independent association with 20-year CVD risk even in the fully adjusted model (i.e., adjusted for age, sex, hypercholesterolemia, hypertension, diabetes, abnormal waist circumference, smoking habits, Mediterranean diet adherence and physical activity). Among the lifestyle factors, adherence to the Mediterranean diet was independently associated with lower 20-year CVD risk in all models. However, among the clinical factors, hypercholesterolemia, hypertension, diabetes, and abnormal waist circumference were consistently associated with increased 20-year CVD risk. As a result, the attributable CVD risk fraction increased from 43% in the core, i.e., age- and sex-adjusted model, to 56% when clinical and anthropometric characteristics (i.e., hypercholesterolemia, hypertension, diabetes, abnormal waist circumference) were considered and, finally, to 86% when trajectories of lifestyle habits were added (Table 4). Being physically active or close to a Mediterranean diet throughout the study’s course (i.e., lifespan) were the most beneficial trajectories against CVD risk. At the same time, persistently smoking was the most detrimental. Moreover, participants who were initially close to the Mediterranean diet but changed to an unhealthier pattern were still protected against CVD development. However, quitting smoking or staring engaging in physical activities during the 20-year observation did not offer significant protection (Table 4).

## 4. Discussion

The present study aimed to assess CVD morbidity and mortality and its related cardio-metabolic risk factors in Greece and to evaluate the association between three major lifestyle trajectories, i.e., smoking habits, Mediterranean diet adherence, and physical activity level, with CVD incidence.

### 4.1. CVD Epidemiology in Greece

The global epidemiology of CVD has received much attention recently, as CVDs are still the leading cause of morbidity and mortality worldwide. The current 2021 ESC Guidelines for CVD prevention [7] and the Global Burden of Disease project [17] classified Greece as a moderate CVD risk country instead of a low risk that was thought for decades (i.e., since the 1950s and the legendary Seven Countries Study). During a 20-year follow-up period of our study, it was observed that almost one-third of the middle-aged (at baseline examination in 2002) participants developed a fatal or non-fatal CVD event. Unfortunately, no other study regarding the Greek population has evaluated CVD incidence, so comparisons cannot be made. In addition, if the observed in this study CVD incidence is extrapolated to the total Greek adult population, it could be speculated that approximately 2 million adults (18–90 years) in Greece have developed any form of CVD during the past 20 years. Thus, contrary to what was previously thought, it is supported that Greece is no longer a low CVD incidence country but a moderate-to-high-risk. However, mortality due to CVD in Greece is still low, as it was observed that no more than 5% of CVD events were fatal. Concurrent epidemiologic data in Europe reflect similar trends. Specifically, during the last 30 years, CVD incidence in Europe has significantly declined by approximately 25%, especially in high-income countries. Notwithstanding, the CVD burden still has a devastating impact on European population health, with enormous financial consequences [18].

In addition, as it was observed here, the man-to-woman ratio was 1.25, with the peak difference observed in the 35–45 age group (i.e., 2.1), but, notably, a reversal of the CVD incidence sex ratio was observed in the group between 55–75 years where women exceeded men, with a resumption of an almost equal incidence after >75 years. Thus, CVD may no longer be considered a “men-hypothesis” as believed in the past.

It is also important to underline that a substantial percentage (9%) of young participants of the ATTICA Study (i.e., <45 years old at baseline) developed a CVD event during the 20-year observation period. Although young heart patients have attracted research interest in the past years, robust evidence about CVD incidence and its determinants in young people is lacking [18].

It was also observed that the prevalence of the majorCVD-related clinical risk factors, hypercholesterolemia, diabetes, and hypertension, increased independently of the aging of the sample, which can be attributed to several other factors, too; for example, the adoption of unfavourable lifestyle behaviors, like sedentarism, unhealthy diets, and smoking, the lack of adherence to medication or untreated conditions, and limited access to healthcare, especially because of the 10-year financial crisis and austerity Greece has faced [2,19,20,21,22,23].

### 4.2. Lifestyle Factors in Relation to CVD Incidence

Another main goal of this study was to assess lifestyle trajectories and their role in long-term CVD development. Based on the present analysis, specific lifestyle trajectory patterns regarding smoking, dietary habits, and physical activity were studied. As revealed, participants were more likely to quit smoking during the last ten years of the follow-up. In contrast, relatively few had started, something that could be attributed to campaigns to reduce smoking implemented in the country in the past years [24] or the aging of the cohort; dietary habits remained mainly unchanged, which is also supported by previous studies that have highlighted the stability of dietary habits, especially among middle-aged or older adults [25]; and physical activity levels slightly decreased, which can be explained by the aging of the studied sample, as it has been observed in other studies, too [17,19]; it is also notable that the prevalence of low (inadequate) physical activity was high, and similar in all examinations of the study’s participants. Notably, the studied lifestyle trajectories accounted for a considerable proportion of CVD incidence during the last 20 years, as the attributable risk was 30%. Specifically, based on the nested models (see Table 4), smoking habits trajectories accounted for 22% of the attributable CVD risk. In comparison, dietary and physical activity trajectories accounted for an additional 4% of the attributable CVD risk, respectively.

Cigarette smoking habit is considered one of the most important threats to human health. Its role in CVD development is now well-established in numerous studies. In the present study, smoking trajectory had the most critical role in CVD development among all three lifestyle factors studied. It is also interesting that smoking quitters, during the 20-year follow-up, did not show any significant protection against CVD development. Accumulated evidence shows that smoking has irreversible effects on the genetic material of humans and can also change the epigenetic landscape [26]. To completely eradicate the observed persistence of increased CVD risk, even in those that stopped smoking, public health actions should focus on helping individuals, especially young ones, to never start smoking.

Regarding dietary habits, it is understood and appreciated that the Mediterranean diet offers a substantial cardioprotective effect. In a previous analysis of the ATTICA Study, it was observed that greater adherence to a Mediterranean type of diet confers a considerable and independent of other lifestyle factors reduction on CVD risk during the first ten years of follow-up, suggesting that even subjects with other unhealthy lifestyle behaviors may benefit from adherence to this diet [27,28]. Based on the present analysis, the previous findings have been expanded, i.e., being close to the Mediterranean diet was protective against CVD risk, even if this adherence was not sustained throughout the study course. Therefore, emphasis should be given to introducing adherence to a healthy diet as early as possible. It is noted that this decline in Mediterranean diet adherence has been observed in several other studies, too [29], and could be possibly explained by a prolonged nutrition transition phenomenon (i.e., worsened dietary habits due to urbanization or certain socio-economic factors) [30].

It is also well-established that physical activity offers significant protection against CVD development. The most recent guidelines of the European Society of Cardiology (2021) suggest that adults of all ages should be engaged in moderate-to-high-intensity exercise at least three times per week to reduce CVD mortality and morbidity [7]. Our study revealed that only participants who remained physically active during the entire 20-year period experienced a significant CVD risk reduction (Table 4), underlying the need for continuous efforts to keep a physically active lifestyle throughout the life course.

### 4.3. Actions Needed to Reduce CVD Rates at the Population Level

The beneficial effects of improving healthy lifestyle habits in the long term might not be efficient unless accompanied by other concomitant beneficial improvements such as reducing body weight [31,32]. Hence, lifestyle modifications should be personalized to be preservable in the long term. However, effective public health actions are more complex than just recommending healthy behaviors. For behavioral changes to be sustained, changes should be made not only at the individual level, but the interpersonal, community, social and political environments as well [33,34,35]. To help governments attain these goals, the World Health Organization (WHO), along with the United States Centers for Disease Control and Prevention (US CDC), launched the Global Hearts Initiative in 2016, which aims at reducing tobacco use, dietary salt, industrially produced trans fats and managing CVD and its determinants in primary health care (HEARTS technical package) [36]. Given that the HEARTS package has been implemented by some US countries with promising results, more countries could benefit from applying this program [37].

#### Strengths and Limitations

The present study is the first prospective cohort on CVD epidemiology of this magnitude in Greece, and of the few globally, with a long follow-up period (20 years), multiple waves (baseline examination, 5-, 10- and 20-year follow-ups) and a relatively large sample. The sample was representative of the age-sex distribution of the urban Greek population (which reflects about 70% of the total Greek population), and different CVD determinants were studied. However, some limitations exist. Measurement error is a common limitation of epidemiological studies. However, the applied methodology of the current study was similar to that of other prospective studies. Thus, comparisons can be made. Participants lived in the greater Athens metropolitan area, a mainly urban region; therefore, this sample cannot represent the total Greek population, especially those living in non-urban, rural regions.

Furthermore, the fact that adult participants of all ages were recruited, given that they were free of CVD, had, as a result, a small number of CVD events when compared to studies that mainly recruited older participants (naturally prone to cardio-metabolic diseases); this fact could be considered a limitation, but it could be a strength too, as it better reflects the true effect size of risk factors on CVD outcomes at the population level. In surveys of observational nature, such as this one, reliable data may be of concern. Mainly, regarding stroke, even though established definitions were used to evaluate stroke cases, it is doubtful if all cases were correctly identified (mainly for cases of minor stroke). Hence, there is a chance that some of the 24% of other CVD cases we observed could be attributed to stroke. The very high effect size measure of diabetes on CVD risk (i.e., HRs exceed 5) observed here may be due to an over-fitting of the model, especially because a large proportion of people with diabetes eventually developed CVD, as well as a potential recall bias, because of the retrospective nature of physical assessment of the participants. Moreover, this analysis did not explore the potential modifying role of other factors (e.g., educational level, socio-economic status, psychological health, sleep habits).

## 5. Conclusions

The CVD incidence and mortality observed in this study were largely attributed to modifiable lifestyle risk factors. Although this is already known and well appreciated, the current public health strategies seem insufficient. Thus, a life-course personalized approach that is cost-effective and long-term sustained is needed to prevent CVD burden.

## Figures and Tables

**Table 1:** Distribution of the 20-year incidence of CVD in men and women that participated in the follow-up evaluation of the ATTICA study and stratified by age group (*n* = 1988).

		20-Year CVD Incidence, % (*n*)		Man-to-Woman CVD Incidence Ratio
Age at baseline	n	Men (*n* = 987)	Women (*n* = 1001)	
<35 y	516	4.3% (17)	3.1% (10)	1.4
35–45 y	565	7.1% (28)	3.4% (11)	2.1
45–55 y	521	41% (164)	34% (110)	1.2
55–65 y	226	26% (105)	35% (113)	0.74
65–75 y	101	12% (48)	17% (53)	0.70
75+ y	59	8.8% (35)	7.5% (24)	1.17
Overall	1988	40% (397)	32% (321)	1.25

Abbreviations: CVD: Cardiovascular disease, y: years old.

**Table 2:** CVD incidence and trajectories of conventional CVD risk factors from 2002 to 2022 in the ATTICA study participants (*n* = 1988).

Year of Survey	Baseline (2001–2)	5-Year Follow-Up (2007)	10-Year Follow-Up (2011–12)	20-Year Follow-Up (2022)	*p* for Linear Trend
Anthropometric factors					
Body weight, % Overweight/obese	60	61	57	53	0.09
Clinical factors					
CVD, %	- ^1^	9	16	36	<0.001
Hypertension, %	31	41	50	53	0.04
Hypercholesterolemia, %	43	54	67	74	0.03
Diabetes, %	7	13	24	32	<0.001
Lifestyle factors					
Smoking Habits, % Current smoking	41	40	41	29	0.09
Adherence to the MD, % Poor level of adherence	32	33	30	34	0.54
Physical activity, % Low physical activity level	59	71	77	69	0.43

Variables (categorical) are presented as relative frequencies (percentages). In addition, *p*-values for matched outcomes were derived using the McNemar test, and the linear trend was evaluated. ^1^ All participants were free-of-CVD at baseline according to the study’s entry criteria. % Poor level of MD adherence is considered for those with MedDietScore < 27/55. Abbreviations: CVD: Cardiovascular Disease, MD: Mediterranean Diet.

**Table 3:** Lifestyle, anthropometric and clinical characteristics of the ATTICA study participants according to 20-year incidents of combined (i.e., fatal, and non-fatal) CVD events.

		20-Year Incident CVD Events		
	Overall *n* = 1988	No *n* = 1270	Yes *n* = 718	*p*
Anthropometric characteristics				
Body mass index, kg/m^2^	26 (5)	26 (4)	28 (4)	<0.001
Abnormal waist circumference (≥102 cm for men, ≥88 cm for women), %	52	45	68	<0.001
Clinical characteristics				
Hypertension, %	30	20	51	<0.001
Hypercholesterolemia, %	41	30	65	<0.001
Diabetes, %	6.6	1.4	17	<0.001
Family history of CVD, %	37	36	39	0.210
Lifestyle characteristics and trajectories				
Pack-years of cigarette smoking	497 (501)	375 (340)	703 (633)	<0.001
Smoking Habits (2002–2012), %				<0.001
Never smokers	54	56	50	
Started smoking	7	2	19	
Quitted smoking	23	32	4	
Continued smoking	16	10	28	
Physical Activity (2002–2012), %				<0.001
Inactive	50	46	56	
Remained active	13	13	14	
Became inactive	28	27	29	
Became active	10	14	2	
MedDietScore (0–55)	26 (7)	27 (6)	23 (6)	<0.001
Adherence to the Mediterranean Diet (2002–2012), %				<0.001
Always away	24	13	42	
From away (2002) to Close (2012)	9	5	16	
From Close (2002) to away (2012)	47	52	36	
Always close	20	29	5	

Continuous variables are presented as mean (standard deviation) or median (interquartile range) and categorical as relative frequencies. *p*-values refer to differences between participants who developed incident CVD events and those that did not, derived using the t-test (continuous normally distributed variables), the Mann-Whitney non-parametric test (continuous not normally distributed variables) and the chi-square test (categorical variables)—Abbreviations: CVD: cardiovascular disease.

**Table 4:** Results from Cox proportional hazards models that evaluated demographic, lifestyle, biological and clinical characteristics in relation to 20-year fatal or non-fatal incidence of cardiovascular disease (CVD) in the ATTICA study (*n* = 1988).

Factors	Model 1	Model 2	Model 3	Model 4	Model 5
Age, per 1 year	1.30 (1.27, 1.33)	1.28 (1.25, 1.32)	1.27 (1.23, 1.31)	1.26 (1.23, 1.30)	1.24 (1.21, 1.28)
Sex, men versus women	1.89 (1.41, 2.54)	1.71 (1.22 2.38)	1.61 (1.11, 2.34)	1.50 (1.03, 2.20)	1.51 (1.04, 2.21)
Hypercholesterolemia, yes versus no	-	2.49 (1.86, 3.85)	2.44 (1.81, 3.80)	2.22 (1.65, 3.59)	2.23 (1.66, 3.58)
Hypertension, yes versus no	-	1.87 (1.30, 2.70)	1.85 (1.27, 2.68)	1.86 (1.29, 2.70)	1.87 (1.20, 2.71)
Diabetes, yes versus no	-	5.40 (2.31, 12.9)	5.45 (2.32, 13.0)	5.70 (2.41, 13.0)	5.70 (2.40, 13.1)
Abnormal waist circumference, yes versus no	-	1.04 (1.03, 1.06)	1.03 (1.01, 1.05)	1.03 (1.00, 1.04)	1.03 (1.01, 1.05)
Smoking habits (2002–2022) versus Never smokers					
Started smoking	-	-	1.78 (0.79, 4.03)	1.70 (0.71, 3.95)	1.68 (0.69, 3.93)
Quitted smoking	-	-	1.33 (0.63, 2.84)	1.32 (0.62, 2.83)	1.32 (0.62, 2.83)
Continued smoking	-	-	3.02 (2.13, 4.27)	2.80 (1.91, 4.05)	2.77 (1.61, 4.02)
Mediterranean diet adherence (2002–2022) versus Always away					
From away to close	-	-	-	0.97 (0.68, 1.39)	0.83 (0.50, 1.37)
From close to away	-	-	-	0.21 (0.17, 0.27)	0.61 (0.42, 0.89)
Always close	-	-	-	0.05 (0.03, 0.07)	0.50 (0.27, 0.90)
Physical activity (2002–2022) versus Inactive					
Remained active	-	-	-	-	0.90 (0.82, 0.97)
Became inactive	-	-	-	-	0.93 (0.63, 1.36)
Became active	-	-	-	-	0.82 (0.54, 1.27)
Attributable fraction *	43%	56%	78%	82%	86%

* Each cell represents the HRs and 95%CIs for each factor indicated in each line of the first column, adjusted for all the other factors included in each model (Models 1–5). To calculate attributable CVD risk fraction (AF), the prevalence of each factor is required. Thus, for the continuous variables, the median value (age < or >45, MedDietScore < or >27) or the relevant clinical outcome (i.e., hypertension, abnormal waist circumference) was used to calculate the prevalence of each factor in the sample. Abbreviations: 95%CI: 95%Confidence Interval, CVD: Cardiovascular Disease, HR: Hazard Ratio.

## Data Availability

Data described in the manuscript, code book, and analytic code will be made available upon request to the corresponding author.
